# Supplementary material for: Prevalence and associated factors of suicidal behavior among adolescents in Bahir Dar City, Northwest Ethiopia
Source: PLoS One. 2025 May 9;20(5):e0323387. doi: 10.1371/journal.pone.0323387 (PMC12064027; doi:10.1371/journal.pone.0323387)
Supplement: Attachment — Submitted filename: COMMENTS OF THE MANUSCRIPT Prevalence of Suicide.docx [file pone.0323387.s001.docx]

**COMMENTS OF THE MANUSCRIPT:**

***Prevalence and associated factors of suicidal behavior among***

***adolescents at Bahir Dar city, North West Ethiopia, community based study, 2021***

**ABSTRACT**

*Under methods;* The sentence “To determine the independent components related with suicidal behaviour, bi-variable and multivariable logistic regression were use” should read have “d” added to use - to read: "To determine the independent components related with suicidal behaviour, bi-variable and multivariable logistic regression were used.

**INTRODUCTION**

*Line 4*: In the last sentence, change “it” to It (I to be in upper case).

Do editing review of the write - especially with punctuation, staring letters,

Globally there have been an estimated 804 000 suicide deaths arose in 2012, representing a twelve-monthly global age-standardized suicide rate of 11.4 per 100 000 population (15.0 for males and 8.0 for females) - reframe this sentence and PROVIDE MORE RECENT DATA, 2012 as a standalone data is obsolete, except authors can show a trend. IF SO, CITE THE SOURCE OF THE INFORMATION!

Sub-Saharan Africa there are over 34,000 (IQR 13,141 to 63,757) suicides per year, with an overall incidence rate of 3.2 per 100,000 populations (13) --- THIS INFORMATION IS OBSOLTE FOR A RECENT FACT (10 years ago). UPDATE THE INFORMATION

*Citations* - (13),(14) and (15) are outdated - provide more recent data.

*Comments on Introduction*: UPDATE THE LITERATURE

**METHODS AND MATERIALS**

Under ***Participants****:* Re frame the sentence. Correct the grammar and use formal language and avoid use of words like “couldn’t.”

Under ***Measurements -*** EDIT THE SECTION - Grammar

Under ***Ethical considerations* -** Ethical clearance was obtained from Institutional review board (IRB) of Bahir Dar University, College of Medicine and Health Science - INSERT THE APPROVAL NUMBER/DATE or make reference to it as an appendix,

*Line 3 (last part) -* These sentences: “The aims of the study were explain for the study participants and data were collected after assent and written consent. Adolescents aged 18 years and above were gave informed written consent to participate. Participants under the age of 18 were verbal assent to the study and then written consent was obtained from their parents /guardians/ on their behalf” NEED TO SPECIFICALLY CORRECTED.

**RESULTS**

Line 3 under ***Psychosocial and Substance Related Factors*:** …...14.1% (89) of respondents don't have a good relationship with…..USE FORMAL LANGUAGE

**Under** “Factors Associated with suicidal behavior among Adolescent participant”**, review the first sentence structure -** variables cannot be candidates

Comments on other sections: DO THOROUGH EDITING: LANGUAGE AND STRUCTURE
