# Supplementary material for: Prevalence and associated factors of suicidal behavior among adolescents in Bahir Dar City, Northwest Ethiopia
Source: PLoS One. 2025 May 9;20(5):e0323387. doi: 10.1371/journal.pone.0323387 (PMC12064027; doi:10.1371/journal.pone.0323387)
Supplement: Attachment — Submitted filename: Response to Reviewers.docx [file pone.0323387.s003.docx]

**To: Sharada P Wasti, Ph.D., MSc, MHCM, MA, PGDHCM, PGCert**

**Academic Editor**

**PLOS ONE**

Thank you for considering our manuscript and for arranging for it to be reviewed by two reviewers. We have tried to address your comments and the comments / suggestions from the two reviewers. In the Rebuttal letter, we copy each of the comments in bold and provide the RESPONSE underneath. We provide a clean manuscript file version of the revised manuscript with line numbering and this is uploaded as a separate file labeled 'Clean manuscript final version'. We also provide an edited manuscript with track changes and this is uploaded as a separate file labeled as ‘Edited manuscript with Track changes’. We hope we have satisfactorily addressed all the comments and hope that our paper may now be suitable for publication in your journal.

Best wishes

Abdulbasit Sherfa

Corresponding author

**Editor comment**

**Make the title very clear and concise and suggest removing the year, and the title should be like: Prevalence and associated factors of suicidal behavior among adolescents in Bahir Dar city, Northwest Ethiopia.**

Response: Thank you for your suggestion and we corrected the title of the article in the revised manuscript as follow:

Prevalence and associated factors of suicidal behavior among adolescents in Bahir Dar city, Northwest Ethiopia

**• This is not clear why five email IDs before the correspondent author. Please remove from your revised version all those email IDs in the title page.**

Response: we removed in the revised manuscript

**Abstract section**

**• Background should be succinct and justify the requirements of this research rather than long generic information, which you can talk about with contemporary statistics to back up your argument.**

**Response**: Suicide is a significant public health issue worldwide and the third leading cause of death among 15–19-year-olds. In Ethiopia, while suicidal behaviors among adolescents have a considerable impact, there is limited information on these behaviors, especially in community settings. Therefore, this study aimed to assess the prevalence and associated factors of suicidal behavior among adolescents in Bahir Dar city

• **The methods section should be more focused, i.e., who are your study participants (adolescents, which age group?) and what is your sampling strategy? It should be clearly stated. What validated survey tools have you used to measure the prevalence of suicidal behaviour, which is also missing in this section?**

Response: A community-based cross-sectional study was conducted from May 1 to May 30, 2021, involving 692 adolescents aged 11 to 19 years in Bahir Dar city, Northwest Ethiopia. Mini-International Neuropsychiatric Interview (MINI) was used to assess suicidal behaviors. Participants were selected using a multistage sampling method. Data collection was performed through face-to-face interviews employing semi-structured and standardized questionnaires. For processing and analysis, the acquired data was entered into Epi-Data version 3.1 and exported to Statistical Package for Social Science version 24. To assess the strength of associations between the outcome and predictor variables, a logistic regression analysis model with adjusted odds ratios was applied. A p-value of less than 0.05 was considered statistically significant.

**• Results section should be more succinct; particularly, you can start highlighting the prevalence and followed with the high AOR risk variables first and the lowest at last; that makes the presentation clearer**.

Response: The prevalence of suicidal behavior was found to be 19.8% with 95% CI (16.6, 22.8).

Regarding predictive variables: low social support [AOR = 12.42, CI = 5.95, 25.95], Expressed more anger [AOR = 7.30, CI = 2.51, 21.22], Negative childhood experiences [AOR = 7.11, CI = 3.56, 14.20], Family history of suicidal attempts [AOR = 5.66, CI = 2.44, 13.10], Have no a good relationship with their parents [AOR = 5.59, CI = 2.59, 12.08], Depression [AOR = 5.32, CI = 2.64, 10.70], Stressful life events [AOR = 2.91, CI = 1.45, 4.91], and Interpersonal stress [AOR = 2.17, 95% CI 1.07, 4.40] were significantly increased odds of suicidal behavior among participants.
**• Conclusion should be clearly stated, which should be succinct based on your findings and provide a pragmatic recommendation to address the burden.**

Response: **Conclusion and recommendation:** The prevalence of suicidal behavior among participants was high in this study, influenced by factors such as low social support, high anger expression, negative childhood experience, family history of suicidal attempts, poor parental relationships, stressful life events, and depression. Therefore, it is essential to enhance social support systems, provide mental health resources, and implement community-based interventions focused on improving family dynamics and reducing childhood adversity.

**Introduction section
• The introduction should start with the global and country burdens and justify why this research is crucial today. You can back up your claim with current literature, but the current version highlights statistics from 2012/13, which are too old to claim the importance of this study.
• The first part of the introduction provides an overview of the global and your study country's prevalence/incident rates of suicidal behaviour, and the second section consistently highlights the risk factors of suicidal behaviours among adolescents, which should be succinct and supported by current literature.**

Response: Thank you for your comments and we tried to update our introduction based on current literatures as follow:

Suicide is death caused by an act of self-harm that is intended to be lethal. Suicidal behavior include completed suicide, attempted suicide, and suicidal ideation, which includes suicide-related thoughts, plans, and acts [1]. Suicidal behaviors is a serious global public health issue, accounting for more than 2.4% of the global disease burden by 2020[2]. An estimated 703,000 people died by suicide worldwide in 2019, with low- and middle-income countries (LMICs) accounting for roughly three-fourths of all deaths. Suicide was the 4th and the 3rd leading cause of death for those aged 15 to 29 and 15 to 19, respectively, and nearly 9 out of 10 were from LMICs, which are home to nearly 90% of all adolescents worldwide [3-6]. Africa has six of the ten highest suicide rates in the world, with 11 suicide fatalities per 100,000 people annually [7]. According to WHO (2021), about 34,000 suicides are reported in Africa each year, accounting for approximately 3.2 per 100,000 people [8, 9]. According to pooled data, the prevalence of suicidal ideation and attempted suicide in Ethiopia ranged from 1% to 55%, and 0.6% to 14% [10].

Adolescence is a critical period marked by physical, emotional, and societal changes, in which people might engage in a variety of risky behaviors, including suicidal ideation, suicide attempts, and suicide [11-13]. Suicide is a principal cause of death among adolescents. Self-harm is the most risk factor for suicide, yet the majority of self-harm does not come to the attention of health services [14]. Suicidal behavior, deliberate self-harm, and non-suicidal self-injury are significant precursors of suicide in children and adolescents [15]. Adolescent psychiatric emergencies are a leading cause, with suicidal attempts and deaths being the strongest predictors [16]. Suicidal thoughts and suicidal behaviors develop during adolescence and peak late in adolescence and early adulthood [17].

Worldwide, the possible risk factors of suicide include victimization (bullying, sexual harassment), poor psychological state related to depression, phobic disorders, anxiety, alcohol use disorder, child abuse, impulsivity, and lack of parental understanding [12, 18, 19]. Studies show that mental state issues, substance use, genetic and biological factors, poor physical health, physical disability, and family-environmental factors increase the risk of suicidal behaviors in adolescents [16, 20, 21]. Suicide is a prevalent issue among adolescents, with those who have experienced suicidal ideation and attempts being significantly more likely to commit suicide [22].

Suicide costs billions of dollars in lost productivity and healthcare expenditures, requiring comprehensive prevention initiatives, enhanced mental health services, public awareness campaigns, and community engagement [23]. Suicide prevention requires robust healthcare systems, universal health coverage, and community engagement. WHO's tool kit aims to address stigma, improve knowledge, and provide social support [24]. In 2013, the 66th World Health Assembly adopted the WHO's Mental Health Action Plan, focusing on suicide prevention to reduce suicide rates by 10% by 2020[25].

Adolescent suicidal behavior is a neglected public health issue, especially in LMICs including Ethiopia [26]. Underinvestment on mental health and lack of national health insurance coverage in LMICs make it more difficult to get resources and help, particularly for young people between the ages of 15 and 29, who are faced with trauma, social stigma, and financial difficulties [27]. It is a multipart and multidimensional phenomenon stemming from the interaction of several factors [28]

Suicide rates are increasing rapidly among young individuals, particularly males aged 15-24, and the rate is still growing [10]. Even though LMICs account for the bulk of suicides, high-income Western nations are the source of most of the knowledge about suicidal behaviors. Because there aren't many studies on teenagers in Ethiopia, little is known regarding the prevalence and determinants of suicide behavior (suicidal thoughts, suicidal plans, and suicidal attempts). Therefore, this study aimed to evaluate the prevalence of suicidal behavior and its contributing factors among adolescents.

**Methods and material:**

**• Study design should be succinct and focused on using credible sources.
• Separate the study design and study site; the study site and participants could be put together if you want, and focus your writing very clearly.**

**• Please state sample size and sampling procedure in one section and make data collection in a separate section. Make data collection very clear with what the core tools you have used are and how you validated those tools, which should be succinctly stated.**

**Response:** we corrected methods and material section as follow:

**Study setting**

The study was conducted in Bahir Dar city Northwest Ethiopia which is 565 kilo meters far from Addis Ababa. According to Bahir Dar city mayor office report in 2019/2020, the estimated population in the city was 373,073. From the total population the number of adolescent (between 10-19 years) was 84,382. The city has one comprehensive specialized hospital, one primary hospital, one specialized public hospital and four private hospitals, which give service to the city and surrounding population. However, only the comprehensive specialized and specialized hospitals have inpatient psychiatric service [29, 30].

**Study design and period**

A community-based cross-sectional study was conducted in Bahir Dar city Northwest Ethiopia from May1-30, 2021.

**Study participants**

All adolescents (aged 10-19 years old) residing in Bahir Dar city were source population whereas adolescents who had been living for the last six months within the selected sub cities of the town were study population. Moreover, households were taken as sampling unit and selected eligible adolescent was considered as study unit. Adolescents who were residing for last six months in Bahir Dar city selected sub cities were included and adolescents from selected households who were severely ill and unable to respond to the interviewer properly were excluded.

Sample size determination and sampling methods

Sample size was determined using a single population proportion formula using the proportion of suicidal behavior 22.5% from previous study in Ethiopia [31] and 95% confidence interval was used, 4% margin of error, design effect 1.5 and by assuming a 10% non-response rate, the final sample size was 692.

Multistage sampling technique was used to select the sub-cities (three) from the total of six and to select respective administrative kebeles (the smallest administrative unit) (six) from total of seventeen. The households in the administrative kebeles were selected by systematic random sampling technique after identifying an initial starting household by use of random number. Eligible adolescents in the selected household were further selected and interviewed. Only one adolescent member of the house hold was selected by lottery method for the interview towards suicide behavior

Data collection

The questionnaire included items to assess socio-demographic information, suicidal behavior, self-esteem, social support, adverse childhood experience, anger expression, stressful life events, clinical factors, behavioral factors and interpersonal stress. Three health extension workers collected the data using face-to-face interviews. The data collection was supervised by a psychiatry nurse (bachelor’s degree holder). Training on the questionable and data collection proceed was provided for the supervisor and data collectors.

**Measurements**

**Suicidal behaviors:** Participants who answered "yes" to at least one of the six questions in the Mini-International Neuropsychiatric Interview (MINI) were categorized as displaying suicidal behavior. The MINI shows high internal consistency (Cronbach’s alpha = 0.84) [32].

**Social support:** The participants' social support was assessed using the Oslo-3 social support scale (25). Based on their scores, participants were classified as follows: those with scores between "3–8" were considered to have poor social support, scores of "9–11" indicated moderate support, and scores ranging from "12-14" represented strong social support.

**Depression**: Depression was assessed by a nine-item Patient Health Questionnaire-9, which has four response categories referring to the amount of time that the symptom was present (not at all (0), several days (1), more than half of the days (2), nearly every day (3)), with a total score ranging from 0 to 27 [33]. Adolescents who scored ≥5 in PHQ-9 were considered as having depression [34]

**Adverse child hood experience:** The ACE Questionnaire (Adverse Childhood Experiences Questionnaire) was used to assess the impact of traumatic childhood experiences, including abuse, neglect, and household dysfunction. It consists of questions about various adverse experiences [35].

**Self-esteem:** The participant's self-esteem was assessed using the Rosenberg self-esteem scale. People scoring between 15 and 25 are average. A score of less than 15 suggests low self-esteem and a score greater than 25 suggests high self-esteem [36].

**Anger Expression:** The participant's expression of anger was evaluated using the Spielberger Anger-Out Expression Scale, which includes eight items designed to assess the coping strategies individuals employ, especially in relation to outward expressions of anger. This scale categorizes anger expression into three levels: low anger expression (scores below 10), moderate anger expression, and high anger expression (scores of 15 or above) [37].

**Stress full life events:** According to the SLESQ definition, individuals who reported a positive response to at least one of the eleven specific categories or the two general categories of events—such as enduring a life-threatening accident, experiencing physical or sexual abuse, or witnessing a murder or assault—were classified as having undergone stressful life events [38].

**Interpersonal stress:** Interpersonal stress was asses by using Bergen Social Relationships Scale BSRS. The scale consists of six items. Individuals with high scores (above the mean) of BSRS have high interpersonal stress [39].

**Fear of corona virus**. The assessment of fear associated with the coronavirus was carried out using a specially designed seven-item scale. Participants indicated their level of agreement with a range of statements using a five-point Likert scale, which included the options "strongly disagree," "disagree," "neutral," "agree," and "strongly agree". Individuals with high scores (above the mean) have a fear of coronavirus [40].

Clinical variables like family history of suicidal attempts and other factors like growing up with their parent were assessed by asking the participants

**• Make an "analysis" section with data analysis. State very clearly why you used (P ≤ 0.20) in the bivariate analysis instead of p < 0.05 in the multivariate logistic regression analysis.**

Response: thank you for your suggestion and we tried to make analysis section as data processing and analysis and we clearly stated the reason why we used why you used (P ≤ 0.20) in the bivariate analysis as follow:

The reason for using a p-value threshold of less than 0.2 or 0.25 in binary logistic regression for variable selection allows for a more exploratory approach, enabling the identification of potentially relevant predictors that may be clinically or theoretically significant, even if they don't meet the stricter p < 0.05 criterion. The other thing is this higher threshold helps mitigate Type II errors by reducing the risk of excluding important variables, particularly in complex models where multicollinearity or interaction effects may obscure significance.

Data processing and analysis

The data was entered into Epi data version 3.1 and exported to SPSS version 24 for analysis. Necessary data processing like recoding, categorizing, merging, computing, and counting was done before the actual data analysis. After data processing, descriptive statistics like measures of central tendency (mean, median, and mode) and measures of dispersion were used for continuous variables and frequency count and proportion were used to summarize categorical variables. All variables with a p-value of less than 0.2 in the bivariate logistic regression analysis were entered into the multivariable logistic regression model to identify factors associated with suicidal behavior. The adjusted odds ratios (AORs) with 95% confidence intervals were used to assess the strength of associations between the outcome and predictor variables. The p-value of <0.05 was considered significant.

**Ethical approval and consent to participate**

Ethical clearance was obtained from Institutional review board (IRB) of Bahir Dar University, College of Medicine and Health Science [REF.178/2021]. Then letter of permission was obtained from Bahir Dar city administration health office and permission was granted from the city administrator. The aims of the study were explained to the participants, and data were collected after obtaining assent and written consent. Adolescents aged 18 years and above provided informed written consent to participate. Participants under the age of 18 gave verbal assent to the study, and written consent was then obtained from their parents or guardians on their behalf. Both adolescents and parents were informed that they had the right to refuse in answering any question at any time. Referral was given for participants who had suicidal behavior and depressive symptoms

**Results section**

**• Make the socio-demographic characteristic section narration very succinct and also add the mean and SD age in Table 1.**

Response: A total of 631 adolescents participated in this study, yielding a response rate of 91.2%. The average age of the participants was 16.1 years (SD = 2.0), with 99.4% being unmarried. Most respondents (83.7%) resided with their parents, while 6.5% lived with others, and 9.8% had independent living situations. Over half of the participants (55.2%) identified as female

**• Clinical and other related factors not very clear how you measured depression and fear of COVID-19; which tools should you have used that should be clearly stated in the data collection tools section?**

Response: Thank you for your comments and we clearly stated those tools how to measure depression and fear of covid 19 in the revised manuscript as follow:

Depression was assessed by a nine-item Patient Health Questionnaire-9, which has four response categories referring to the amount of time that the symptom was present (not at all (0), several days (1), more than half of the days (2), nearly every day (3)), with a total score ranging from 0 to 27 [33]. Adolescents who scored ≥5 in PHQ-9 were considered as having depression [34]

**Fear of corona virus**. The assessment of fear associated with the coronavirus was carried out using a specially designed seven-item scale. Participants indicated their level of agreement with a range of statements using a five-point Likert scale, which included the options "strongly disagree," "disagree," "neutral," "agree," and "strongly agree". Individuals with high scores (above the mean) have a fear of coronavirus [40]

Out of the total participants, 121 individuals, or 19.2%, were found to be experiencing depression. Regarding stressful life events, 40.9% of participants reported having encountered such situations. Additionally, more than one-third (36.3%) indicated that they had faced adverse childhood experiences. Concerning self-esteem, 24.6% of respondents reported low self-esteem, while 60.4% reported moderate self-esteem, and 6% reported high self-esteem. Moreover, 62.4% of participants displayed moderate anger expression behaviors, with 13.8% showing higher levels of anger expression. Approximately 60% of participants expressed a fear of COVID-19, and 51.5% reported experiencing high levels of interpersonal stress.

**• Please improve your presentation style, which is not good to start academic writing in percentage, i.e., 34%, 53.6% in the quantitative presentation.**

Response: Thank you for your comments and we tried to improve in the revised manuscript as follow:

The study revealed a prevalence of suicidal behavior at 19.8% (125 individuals), with a 95% confidence interval ranging from 16.6 to 22.8. Almost half (48%) of the participants were late adolescents (ages 18–19), and a significant majority (71.2%) were female. Furthermore, 34% reported a family history of suicide attempts, 85% had experienced adverse childhood events, and 22.4% had a history of alcohol use. Almost half (48%) indicated poor relationships with their parents, while 76% reported experiencing stressful life events. Regarding anger expression, 53.6% displayed moderate levels, and 33.6% showed high levels. For details on gender distribution and anger expression, refer to Figures 1 and 2.

**• Make very clear of the “factors associated with suicidal behaviour among adolescent participants” section, which should start presenting your findings from the highest AOR to the lowest one.**

Response:

Thank you for your comments and we corrected in the revised manuscript and reorganizing factors associated with suicidal behavior from highest AOR to lowest one as follow:

In bi-variable logistic regression Age, sex, living status, ever use of alcohol, family history of suicidal attempts, growing up with a parent, relationship with a parent, social support, adverse childhood experience, stressful life experience, anger out expression behavior, depression, interpersonal stress, and current use of tobacco were candidates for multiple variable regression at p < 0.2. In the multivariate analyses, family history of suicidal attempts, facing stressful life experiences, having adverse childhood experiences, having poor social support, not having a good relationship with their parents, depression, interpersonal stress, and having high anger expression behaviors were found to be significantly associated with suicidal behavior at p  < 0.05.

Adolescents with insufficient social support were approximately 12.42 times more likely (AOR = 12.42, CI = 5.95–25.95) to report suicidal behavior compared to their peers who had strong social support. Additionally, those who exhibited high levels of anger expression had 7.3 times the odds of reporting suicidal behavior (AOR = 7.30, CI = 2.51-21.22) compared to individuals with lower anger expression. Furthermore, adolescents who experienced adverse childhood events, such as abuse, neglect, and household dysfunction, faced a sevenfold increase in the risk of suicidal behavior (AOR = 7.11, CI = 3.56–14.20) compared to their peers.

Moreover, adolescents with a family history of suicide attempts had a 5.66 times higher risk (AOR = 5.66, CI = 2.44, 13.10) of exhibiting suicidal behavior than those without such a background. Participants who reported poor relationships with their parents also showed increased odds (AOR = 5.59, CI = 2.59–12.08) of developing suicidal behavior compared to those with positive parental relationships. Those suffering from depression were about five times more likely (AOR = 5.32, CI = 2.64–10.70) to engage in suicidal behavior. Similarly, adolescents facing stressful life experiences had higher odds (AOR = 2.91, CI = 1.45–4.91) of suicidal behavior compared to their peers. Finally, participants experiencing high levels of interpersonal stress were approximately twice as likely (AOR = 2.17, 95% CI = 1.07–4.40) to report suicidal behavior compared to those with lower levels of interpersonal stress.

**Discussion Section:**

**• Please organise your discussion section into two parts: 1) Prevalence of suicidal behaviours and 2) factors associated with the suicidal behaviours. Each section starts with your findings first, and that should be compared and contrasted with the global literature and provide your thoughts if particular findings are unique or already reported from literature.**

Response: Thank you for your comment; we organized the discussion as suggested on the revised manuscript as follows

The main objective of this study was to assess the prevalence and factors associated with suicidal behavior among adolescents in Bahir Dar city. The prevalence of suicide behavior was 19.8% (95% CI: 16.6-22.8). This finding was consistent with a study conducted in Ethiopia at Dangila Town, which showed that 16.2% had attempted suicide and 22.5% had suicidal ideation [31]; Ghana that reported suicidal behaviors: 18.2%, 22.5%, and 22.2% for suicidal ideation, suicidal plan, and suicidal attempt, respectively [41]; Mozambique (17.7% suicidal ideation and 18.5% suicidal attempt) [42]; and Togo (16.5% suicidal thought) [43]. Prevalence on the current finding was higher than a school-based study done in Kut City, which reported suicidal behavior to be 8.3% (44) and Tunisia (9.6% suicidal ideation and 7.3% suicidal attempt) [44] and Tunisia (9.6% suicidal ideation and 7.3% suicidal attempt) [45]. In contrast, it was lower than studies done in Liberia, where the prevalence of suicidal attempt and suicidal ideation were 33.7% and 26.8%, respectively [46]; Lebanese 28.9% suicidal ideation [12]; and Benin (23.2% suicidal thought and 28.3% suicidal attempt) [21]. The discrepancy might be due to variations in respondents' socio-demographic, economic, and cultural characteristics; sample size; study setting; and measurement tools like the MINI Suicidal Scale [32].

In the multivariate analyses, factors significantly associated with suicidal behavior include family history, stressful life experiences, adverse childhood experiences, poor social support, poor parent-child relationships, depression, and high anger-out expression behavior. According to this study, adolescents with low social support had a 12.42 times higher risk of suicide behavior than those with good social support [AOR=12.42, CI=5.95, 25.95]. Previous research has also revealed that low social support is linked to suicide behavior [2, 31, 47]. Adolescents who did not have a good relationship with their parents were 5.59 times more likely to engage in suicide conduct than those who did [AOR=5.59, 95% CI (2.59, 12.08). The findings of the study were supported by prior research conducted in China and Japan [16, 48]. The possible explanation might be that adolescents with low social support can lead to feelings of isolation and hopelessness, which are risk factors for suicidal behavior, and a supportive family environment can provide emotional stability and resilience against mental health issues.

The current study discovered that adolescents with higher anger expression behavior had approximately seven times (AOR=7.30, CI= 2.51-21.22) the odds of reporting suicide conduct as those with lower anger expression behavior. The possible reason may be individuals with anger-related disorders exhibit a range of cognitive and interpersonal deficiencies that have a negative impact on well-being and contribute to a higher risk for suicide behaviors, such as poor social problem-solving abilities and poor social reasoning. The findings of this study lend support to the concept that high levels of external rage emotions are significantly related with depressive symptoms and suicidal conduct.

Adolescent having adverse child-hood experience (abuse, neglect and household dysfunction) had seven fold (AOR=7.11, CI= 3.56-14.20) higher risk of having suicidal behavior compared to their counterparts previous studies also found that adolescent those experiencing abuse, physically hurt and experiencing violence is positively associated with suicidal behavior [35]. Participants who had to experiencing stressful life events 2.91 times the odds of suicidal behavior than their counterparts [(AOR= 2.91, 95% CI=1.43, 5.90)]. This finding was supported by previous Studies in which suicidal behavior was significantly favored by stressful life events [45, 49]. Adverse childhood experiences and stressful life events can lead to lasting psychological effects, including increased vulnerability to mental health issues like depression and anxiety, or can trigger or worsen existing mental health issues, leading to increased suicidal thoughts and behaviors.

Adolescents with a family history of suicidal conduct were 5.66 times more likely to attempt suicide than adolescents without such a history [AOR = 5.66, 95% CI (2.44, 13.10)]. This finding was supported by study done in Fitche Town, north Shoa, Oromia region [47], but no significant associations was observed in the study conducted in Dangila town [31]. The discrepancy could be attributed to the study subjects' coping mechanisms, as well as the sample size and study context.

Participants with depression showed a fivefold higher risk of suicide conduct [AOR = 5.32, 95% CI = 2.64, 10.70] than those without depression. Previous studies [22, 44, 50], also have found adolescent had depression is positively associated with suicidal behaviors. Participants who had high interpersonal stress had approximately 2 times the odds [AOR=2.17, 95% CI (1.07.4.40)] of suicidal behavior compared to those who had low interpersonal stress. Previous study also support that high interpersonal stress was significantly associated with suicidal behavior [51].The possible reason for this might be Stress in interpersonal domains, meaning stress that occurs in the context of relationships with others, has consistently been identified as a significant correlate or predictor of suicidal behavior in adolescence.

**Limitation section:**

**• Please also add the strengths of the study with limitations.**

Response: we add strengths of the study in the revised manuscript

While the use of standard and validated tools to assess both dependent and independent variables and taking a sufficient sample to represent the target population were considered the strength of the study, this study is not without its limitations. Because it is cross-sectional causal relationships cannot be established between independent factors and suicidal behaviors. Information bias may occur during data collection. This study did not account for the presence of psychiatric or psychological disorders other than depression, such as schizophrenia, which has been associated with suicidal behavior. This study was conducted in one city in Ethiopia, which limits its generalizability to other settings.

**Conclusion section:**

**• Draw your conclusion very clearly, concisely, and coherently. What were the key outcomes of your study? Provide a pragmatic suggestion in a paragraph rather than so many subheadings that should be clear, concise, and coherent.**

Response: Thank you for your comments and we tried to make our conclusion clear and concise based on our outcome in the revised manuscript as follow:

# Conclusion and Recommendation

The prevalence of suicide behavior was rather high in this study, indicating a substantial public health risk among teenagers in Bahir Dar that demands special attention. Suicidal behaviour was significantly associated with a lack of social support, depression, a family history of suicide, not having a good relationship with their parent, adverse childhood experiences, and experiencing stressful live events**.**

As a result, a collaborative approach to mental health education and support is essential, which involves working with professionals, encouraging open communication, and establishing strong relationships. Furthermore, improving public awareness of risk factors for suicidal behavior, urging local authorities to adopt effective suicide prevention initiatives, and doing additional research are required.

**General comments:**

**• Thoroughly proofread your entire report and make the consistency of your subheadings’ capital and small letters and the presentation of AOR in all sections.**

**• Thoroughly review the reference list and make your reference list complete and consistent**

Response: Thank you for your comments and we tried to correct the entire manuscript.

**Reviewer 1**

**Reviewer #1: It is an interesting topic. I have reviewed your manuscript and raised some concerns that need a response from you.**

**1. Abstract: rewrite the result section in the abstract in more summarized way. No need of interpretation in this section.**

**2. Methods and materials: In the exclusion criteria, participants participants couldnot communicate well were not included? What does mean by participants couldnot commnunicate well? How many of the participants found to have this problem? I think justify it or avoid it. Additionally, how many of the participants found to have severe illnesses and excluded from the study?
Data quality control: you have missed it in your manuscript, write how you assure to the data quality?**

Response: Thank you for your comments and we tried to rewrite the abstract section in the revised manuscript as follow:

**Background:** Suicide is a significant public health issue worldwide and the third leading cause of death among 15–19-year-olds. In Ethiopia, while suicidal behaviors among adolescents have a considerable impact, there is limited information on these behaviors, especially in community settings. Therefore, this study aimed to assess the prevalence and associated factors of suicidal behavior among adolescents in Bahir Dar city.

**Methods**: A community-based cross-sectional study was conducted from May 1 to May 30, 2021, involving 692 adolescents aged 11 to 19 years in Bahir Dar city, Northwest Ethiopia. Mini-International Neuropsychiatric Interview (MINI) was used to assess suicidal behaviors. Participants were selected using a multistage sampling method. Data collection was performed through face-to-face interviews employing semi-structured and standardized questionnaires. For processing and analysis, the acquired data was entered into Epi-Data version 3.1 and exported to Statistical Package for Social Science version 24. To assess the strength of associations between the outcome and predictor variables, a logistic regression analysis model with adjusted odds ratios was applied. A p-value of less than 0.05 was considered statistically significant.

**Results:** The prevalence of suicidal behavior was found to be 19.8% with 95% CI (16.6, 22.8).

Regarding predictive variables: low social support [AOR = 12.42, CI = 5.95, 25.95], Expressed more anger [AOR = 7.30, CI = 2.51, 21.22], Negative childhood experiences [AOR = 7.11, CI = 3.56, 14.20], Family history of suicidal attempts [AOR = 5.66, CI = 2.44, 13.10], Have no a good relationship with their parents [AOR = 5.59, CI = 2.59, 12.08], Depression [AOR = 5.32, CI = 2.64, 10.70], Stressful life events [AOR = 2.91, CI = 1.45, 4.91], and Interpersonal stress [AOR = 2.17, 95% CI 1.07, 4.40] were significantly increased odds of suicidal behavior among participants.
**Conclusion and recommendation:** The prevalence of suicidal behavior was high in this study showing a significant public health issue among adolescents. Health professionals and both governmental and non-governmental officials should give undue attention in addressing this impacting problem.

Response: In the method and material section we try to rewrite the study participant inclusion and exclusion criteria as follow:

**Study participants**

All adolescents (aged 10-19 years old) residing in Bahir Dar city were source population whereas adolescents who had been living for the last six months within the selected sub cities of the town were study population. Moreover, households were taken as sampling unit and selected eligible adolescent was considered as study unit. Adolescents who were residing for last six months in Bahir Dar city selected sub cities were included and adolescents from selected households who were severely ill and unable to respond to the interviewer properly were excluded.

**Data quality control**

To ensure the data quality, the questionnaire was first developed in English, translated into Amharic language, and translated back into English by different experts to check its consistency. The data was collected using interviewer administered questionnaire from the selected household by three health extension health workers, one BSc psychiatry nurse supervisor, and the principal investigator engaging in the supervision.

The data collectors and the supervisor were given one-day training by the principal investigator. Pre-test was carried out on 5% of the respondents (35 adolescents) in Woreta town and according to the pre-test there was no need of a modification for the questionnaire. The supervisor and principal investigator were closely following the day-to -day data collection process for completeness, clarity and consistency on a daily basis.

**Reviewer 2**

**ABSTRACT**

***Under methods;* The sentence “To determine the independent components related with suicidal behaviour, bi-variable and multivariable logistic regression were use” should read have “d” added to use - to read: "To determine the independent components related with suicidal behaviour, bi-variable and multivariable logistic regression were used.**

Response: we rewrite this section in the revised manuscript as follow:

A community-based cross-sectional study was conducted from May 1 to May 30, 2021, involving 692 adolescents aged 11 to 19 years in Bahir Dar city, Northwest Ethiopia. Mini-International Neuropsychiatric Interview (MINI) was used to assess suicidal behaviors. Participants were selected using a multistage sampling method. Data collection was performed through face-to-face interviews employing semi-structured and standardized questionnaires. The collected data were processed and analyzed using Epi-Data version 3.1 and then exported to the Statistical Package for Social Sciences (SPSS) version 24 for further analysis. To assess the strength of associations between the outcome and predictor variables, a logistic regression analysis model with adjusted odds ratios was applied. A p-value of less than 0.05 was considered statistically significant.

**INTRODUCTION**

***Line 4*: In the last sentence, change “it” to It (I to be in upper case).**

**Do editing review of the write - especially with punctuation, staring letters,**

**Globally there have been an estimated 804 000 suicide deaths arose in 2012, representing a twelve-monthly global age-standardized suicide rate of 11.4 per 100 000 population (15.0 for males and 8.0 for females) - reframe this sentence and PROVIDE MORE RECENT DATA, 2012 as a standalone data is obsolete, except authors can show a trend. IF SO, CITE THE SOURCE OF THE INFORMATION!**

**Sub-Saharan Africa there are over 34,000 (IQR 13,141 to 63,757) suicides per year, with an overall incidence rate of 3.2 per 100,000 populations (13) --- THIS INFORMATION IS OBSOLTE FOR A RECENT FACT (10 years ago). UPDATE THE INFORMATION**

***Citations* - (13), (14) and (15) are outdated - provide more recent data.**

***Comments on Introduction*: UPDATE THE LITERATURE**

Response: we tried to update the literature in the introduction section of the revised manuscript as follow:

Suicide is death caused by an act of self-harm that is intended to be lethal. Suicidal behavior include completed suicide, attempted suicide, and suicidal ideation, which includes suicide-related thoughts, plans, and acts [1]. Suicidal behaviors is a serious global public health issue, accounting for more than 2.4% of the global disease burden by 2020[2]. An estimated 703,000 people died by suicide worldwide in 2019, with low- and middle-income countries (LMICs) accounting for roughly three-fourths of all deaths. Suicide was the 4th and the 3rd leading cause of death for those aged 15 to 29 and 15 to 19, respectively, and nearly 9 out of 10 were from LMICs, which are home to nearly 90% of all adolescents worldwide [3-6]. Africa has six of the ten highest suicide rates in the world, with 11 suicide fatalities per 100,000 people annually [7]. According to WHO (2021), about 34,000 suicides are reported in Africa each year, accounting for approximately 3.2 per 100,000 people [8, 9]. According to pooled data, the prevalence of suicidal ideation and attempted suicide in Ethiopia ranged from 1% to 55%, and 0.6% to 14% [10].

Adolescence is a critical period marked by physical, emotional, and societal changes, in which people might engage in a variety of risky behaviors, including suicidal ideation, suicide attempts, and suicide [11-13]. Suicide is a principal cause of death among adolescents. Self-harm is the most risk factor for suicide, yet the majority of self-harm does not come to the attention of health services [14]. Suicidal behavior, deliberate self-harm, and non-suicidal self-injury are significant precursors of suicide in children and adolescents [15]. Adolescent psychiatric emergencies are a leading cause, with suicidal attempts and deaths being the strongest predictors [16]. Suicidal thoughts and suicidal behaviors develop during adolescence and peak late in adolescence and early adulthood [17].

Worldwide, the possible risk factors of suicide include victimization (bullying, sexual harassment), poor psychological state related to depression, phobic disorders, anxiety, alcohol use disorder, child abuse, impulsivity, and lack of parental understanding [12, 18, 19]. Studies show that mental state issues, substance use, genetic and biological factors, poor physical health, physical disability, and family-environmental factors increase the risk of suicidal behaviors in adolescents [16, 20, 21]. Suicide is a prevalent issue among adolescents, with those who have experienced suicidal ideation and attempts being significantly more likely to commit suicide [22].

Suicide costs billions of dollars in lost productivity and healthcare expenditures, requiring comprehensive prevention initiatives, enhanced mental health services, public awareness campaigns, and community engagement [23]. Suicide prevention requires robust healthcare systems, universal health coverage, and community engagement. WHO's tool kit aims to address stigma, improve knowledge, and provide social support [24]. In 2013, the 66th World Health Assembly adopted the WHO's Mental Health Action Plan, focusing on suicide prevention to reduce suicide rates by 10% by 2020[25].

Adolescent suicidal behavior is a neglected public health issue, especially in LMICs including Ethiopia [26]. Underinvestment on mental health and lack of national health insurance coverage in LMICs make it more difficult to get resources and help, particularly for young people between the ages of 15 and 29, who are faced with trauma, social stigma, and financial difficulties [27]. It is a multipart and multidimensional phenomenon stemming from the interaction of several factors [28]

Suicide rates are increasing rapidly among young individuals, particularly males aged 15-24, and the rate is still growing[10]. Even though LMICs account for the bulk of suicides, high-income Western nations are the source of most of the knowledge about suicidal behaviors. Because there aren't many studies on teenagers in Ethiopia, little is known regarding the prevalence and determinants of suicide behavior (suicidal thoughts, suicidal plans, and suicidal attempts). Therefore, this study aimed to evaluate the prevalence of suicidal behavior and its contributing factors among adolescents.

**METHODS AND MATERIALS**

**Under *Participants:* Re frame the sentence. Correct the grammar and use formal language and avoid use of words like “couldn’t.”**

Response: we corrected the grammar and the language in the revised manuscript as follow:

**Study participants**

All adolescents (aged 10-19 years old) residing in Bahir Dar city were source population whereas adolescents who had been living for the last six months within the selected sub cities of the town were study population. Moreover, households were taken as sampling unit and selected eligible adolescent was considered as study unit. Adolescents who were residing for last six months in Bahir Dar city selected sub cities were included and adolescents from selected households who were severely ill and unable to respond to the interviewer properly were excluded.

**Under *Measurements -* EDIT THE SECTION – Grammar**

Response: Thank you for the comments and we tried to check and corrected in the revised manuscript as follow:

**Measurements**

**Suicidal behaviors:** Participants who answered "yes" to at least one of the six questions in the Mini-International Neuropsychiatric Interview (MINI) were categorized as displaying suicidal behavior. The MINI shows high internal consistency (Cronbach’s alpha = 0.84) [32].

**Social support:** The participants' social support was assessed using the Oslo-3 social support scale (25). Based on their scores, participants were classified as follows: those with scores between "3–8" were considered to have poor social support, scores of "9–11" indicated moderate support, and scores ranging from "12-14" represented strong social support.

**Depression**: Depression was assessed by a nine-item Patient Health Questionnaire-9, which has four response categories referring to the amount of time that the symptom was present (not at all (0), several days (1), more than half of the days (2), nearly every day (3)), with a total score ranging from 0 to 27 [33]. Adolescents who scored ≥5 in PHQ-9 were considered as having depression [34]

**Adverse child hood experience:** The ACE Questionnaire (Adverse Childhood Experiences Questionnaire) was used to assess the impact of traumatic childhood experiences, including abuse, neglect, and household dysfunction. It consists of questions about various adverse experiences [35].

**Self-esteem:** The participant's self-esteem was assessed using the Rosenberg self-esteem scale. People scoring between 15 and 25 are average. A score of less than 15 suggests low self-esteem and a score greater than 25 suggests high self-esteem [36].

**Anger Expression:** The participant's expression of anger was evaluated using the Spielberger Anger-Out Expression Scale, which includes eight items designed to assess the coping strategies individuals employ, especially in relation to outward expressions of anger. This scale categorizes anger expression into three levels: low anger expression (scores below 10), moderate anger expression, and high anger expression (scores of 15 or above) [37].

**Stress full life events:** According to the SLESQ definition, individuals who reported a positive response to at least one of the eleven specific categories or the two general categories of events—such as enduring a life-threatening accident, experiencing physical or sexual abuse, or witnessing a murder or assault—were classified as having undergone stressful life events [38].

**Interpersonal stress:** Interpersonal stress was asses by using Bergen Social Relationships Scale BSRS. The scale consists of six items. Individuals with high scores (above the mean) of BSRS have high interpersonal stress [39].

**Fear of corona virus**. The assessment of fear associated with the coronavirus was carried out using a specially designed seven-item scale. Participants indicated their level of agreement with a range of statements using a five-point Likert scale, which included the options "strongly disagree," "disagree," "neutral," "agree," and "strongly agree". Individuals with high scores (above the mean) have a fear of coronavirus [40].

Clinical variables like family history of suicidal attempts and other factors like growing up with their parent were assessed by asking the participants

**Under *Ethical considerations* - Ethical clearance was obtained from Institutional review board (IRB) of Bahir Dar University, College of Medicine and Health Science - INSERT THE APPROVAL NUMBER/DATE or make reference to it as an appendix,**

Response: Thank you for comments and we stated and provide ethical approval with approval number in the declaration section of the manuscript as follow:

Ethical clearance was obtained from Institutional review board (IRB) of Bahir Dar University, College of Medicine and Health Science [REF.178/2021]. Then letter of permission was obtained from Bahir Dar city administration health office and permission was granted from the city administrator. The aims of the study were explained to the participants, and data were collected after obtaining assent and written consent. Adolescents aged 18 years and above provided informed written consent to participate. Participants under the age of 18 gave verbal assent to the study, and written consent was then obtained from their parents or guardians on their behalf. Both adolescents and parents were informed that they had the right to refuse in answering any question at any time. Referral was given for participants who had suicidal behavior and depressive symptoms

***Line 3 (last part) -* These sentences: “The aims of the study were explain for the study participants and data were collected after assent and written consent. Adolescents aged 18 years and above were gave informed written consent to participate. Participants under the age of 18 were verbal assent to the study and then written consent was obtained from their parents /guardians/ on their behalf” NEED TO SPECIFICALLY CORRECTED.**

Response: Thank you for your comments and we corrected in the revised manuscript as follow:

The aims of the study were explained to the participants, and data were collected after obtaining assent and written consent. Adolescents aged 18 years and above provided informed written consent to participate. Participants under the age of 18 gave verbal assent to the study, and written consent was then obtained from their parents or guardians on their behalf

**RESULTS**

**Line 3 under *Psychosocial and Substance Related Factors*: …...14.1% (89) of respondents don't have a good relationship with…..USE FORMAL LANGUAGE**

Response: Thank you for your comments and we tried to correct in the revised manuscript as follow:

The study found that 13.5% of participants had a family history of suicide attempts, while only 0.3% indicated a family history of completed suicide. Almost 80% of the participants were raised by their biological parents. Additionally, 14.1% (89 individuals) characterized their family relationships as poor. In contrast, most participants (99.7%) reported having positive relationships with their peers. Many respondents indicated experiencing moderate levels of social support. The rates of tobacco, alcohol, and chat usage among participants were recorded at 4.4%, 9.2%, and 5.1%, respectively.

**Under “Factors Associated with suicidal behavior among Adolescent participant”, review the first sentence structure - variables cannot be candidates**

Response: Thank you for your comments we were saying that In bi-variable logistic regression, Age, sex, living status, ever use of alcohol, family history of suicidal attempts, growing up with a parent, relationship with a parent, social support, adverse childhood experience, stressful life experience, anger out expression behavior, depression, interpersonal stress, and current use of tobacco were candidates for multiple variable logistic regression at p < 0.2.

The reason for using a p-value threshold of less than 0.2 or 0.25 in binary logistic regression for variable selection allows for a more exploratory approach, enabling the identification of potentially relevant predictors that may be clinically or theoretically significant, even if they don't meet the stricter p < 0.05 criterion. The other thing is this higher threshold helps mitigate Type II errors by reducing the risk of excluding important variables, particularly in complex models where multicollinearity or interaction effects may obscure significance

**Comments on other sections: DO THOROUGH EDITING: LANGUAGE AND**

**STRUCTURE**

Response: we tried to edit and check the entire manuscript, grammar, language and structure in the revised manuscript

**References**

1.	Zarrouq, B., et al., *Suicidal behaviors among Moroccan school students: prevalence and association with socio-demographic characteristics and psychoactive substances use: a cross-sectional study.* BMC Psychiatry, 2015. **15**(1): p. 284.

2.	Abdu, Z., M. Hajure, and D. Desalegn, *Suicidal behavior and associated factors among students in Mettu University, South West Ethiopia, 2019: an institutional based cross-sectional study.* Psychology research and behavior management, 2020. **13**: p. 233.

3.	Thornton, V.J., C.B. Asanbe, and E.g.D. Denton, *Clinical risk factors among youth at high risk for suicide in South Africa and Guyana.* Depression and anxiety, 2019. **36**(5): p. 423-432.

4.	Arensman, E., et al., *Suicide and suicide prevention from a global perspective.* Crisis, 2020.

5.	Life, W.L., *An implementation guide for suicide prevention in countries.* World Health Organization, 2021.

6.	World Health Organization, *Suicide worldwide in 2019: global health estimates*. 2021.

7.	*Reversing suicide, mental health crisis in Africa* 06 October 2022, World Health Organization.

8.	*Global Health Estimates: Leading Causes of Death. WHO Global Health Estimates*. 2021, World Health Organization

9.	Mars, B., et al., *Suicidal behaviour across the African continent: a review of the literature.* BMC Public Health, 2014. **14**(1): p. 606.

10.	Bifftu, B.B., et al., *Prevalence of suicidal ideation and attempted suicide in the general population of Ethiopia: a systematic review and meta-analysis.* Int J Ment Health Syst, 2021. **15**(1): p. 27.

11.	World Health Organization. *Adolescent mental health*. 28 September 2020 [cited 2020 December 1].

12.	Pengpid, S. and K. Peltzer, *Suicide attempt and associated factors among adolescents in five Southeast Asian countries in 2015.* Crisis: The Journal of Crisis Intervention and Suicide Prevention, 2020.

13.	Sousa, C.M.d.S., et al., *Suicidal ideation and associated factors among high school adolescents.* Revista de saude publica, 2020. **54**: p. 33.

14.	McMahon, E.M., et al., *The iceberg of suicide and self-harm in Irish adolescents: a population-based study.* Social psychiatry and psychiatric epidemiology, 2014. **49**(12): p. 1929-1935.

15.	Lim, K.-S., et al., *Global lifetime and 12-month prevalence of suicidal behavior, deliberate self-harm and non-suicidal self-injury in children and adolescents between 1989 and 2018: a meta-analysis.* International journal of environmental research and public health, 2019. **16**(22): p. 4581.

16.	Liu, X., et al., *Prevalence of suicidal behaviour and associated factors in a large sample of Chinese adolescents.* Epidemiology and psychiatric sciences, 2019. **28**(3): p. 280-289.

17.	Strandheim, A., et al., *Risk factors for suicidal thoughts in adolescence-a prospective cohort study: the Young-HUNT study.* BMJ open, 2014. **4**(8).

18.	Smith, L., et al., *Prevalence and correlates of multiple suicide attempts among adolescents aged 12-15 years from 61 countries in Africa, Asia, and the Americas.* J Psychiatr Res, 2021. **144**: p. 45-53.

19.	Geremew, A.B., et al., *Suicide attempt and its determinants among youth in central, north, and west Gondar zones, northwest Ethiopia: Findings from the youth health project.* Front Psychiatry, 2022. **13**: p. 1031402.

20.	Liu, Z.-Z., et al., *Suicidal behaviours among Chinese adolescents exposed to suicide attempt or death.* Epidemiology and Psychiatric Sciences, 2020. **29**.

21.	Randall, J.R., et al., *Suicidal behaviour and related risk factors among school-aged youth in the Republic of Benin.* PLoS one, 2014. **9**(2): p. e88233.

22.	Pandey, A.R., et al., *Factors associated with suicidal ideation and suicidal attempts among adolescent students in Nepal: Findings from Global School-based Students Health Survey.* PloS one, 2019. **14**(4): p. e0210383.

23.	Oladeji, B.D., et al., *The Epidemiology of Suicide and Suicidal Behaviour across the Lifespan in Nigeria: A Systematic Review of the Literature.* West Afr J Med, 2021. **38**(9): p. 817-827.

24.	World Health Organization, *Live life: preventing suicide*. 2018.

25.	World Health organaization, *Preventing suicide: A global imperative.* 2014.

26.	Rudatsikira, E., A.S. Muula, and S. Siziya, *Prevalence and associated factors of suicidal ideation among school-going adolescents in Guyana: results from a cross sectional study.* Clinical Practice and Epidemiology in Mental Health, 2007. **3**(1): p. 1-5.

27.	*Global burden of 369 diseases and injuries in 204 countries and territories, 1990-2019: a systematic analysis for the Global Burden of Disease Study 2019.* Lancet, 2020. **396**(10258): p. 1204-1222.

28.	Gvion, Y. and A. Apter, *Suicide and suicidal behavior.* Public health reviews, 2012. **34**(2): p. 9.

29.	Elefachew, A., et al., *Adolescents' Sexual Reproductive Health Service Utilization and Associated Factors Among Bahir Dar City High School Students, Amhara Region, Ethiopia: A Cross-Sectional Study.* Int J Reprod Med, 2024. **2024**: p. 5367867.

30.	Adigeh, D.T. and B.G. Abebe, *The practice of peri-urban land acquisition by expropriation for housing purposes and the implications: the case of Bahir Dar, Ethiopia.* Urban Science, 2023. **7**(2): p. 41.

31.	Amare, T., et al., *Prevalence and associated factors of suicide ideation and attempt among adolescent high school students in Dangila town, Northwest Ethiopia.* Psychiatry journal, 2018. **2018**.

32.	Roaldset, J.O., O.M. Linaker, and S. Bjørkly, *Predictive validity of the MINI suicidal scale for self-harm in acute psychiatry: a prospective study of the first year after discharge.* Archives of suicide research, 2012. **16**(4): p. 287-302.

33.	Hanlon, C., et al., *Validity of brief screening questionnaires to detect depression in primary care in Ethiopia.* Journal of affective disorders, 2015. **186**: p. 32-39.

34.	Kroenke, K. and R.L. Spitzer, *The PHQ-9: a new depression diagnostic and severity measure*. 2002, Slack Incorporated Thorofare, NJ. p. 509-515.

35.	Murphy, A., et al., *Adverse childhood experiences (ACEs) questionnaire and adult attachment interview (AAI): Implications for parent child relationships.* Child abuse & neglect, 2014. **38**(2): p. 224-233.

36.	Rosenberg, M., *Rosenberg self-esteem scale (RSE).* Acceptance and commitment therapy. Measures package, 1965. **61**(52): p. 18.

37.	Terasaki, D.J., et al., *Anger expression, violent behavior, and symptoms of depression among male college students in Ethiopia.* BMC public health, 2009. **9**(1): p. 1-8.

38.	Green, B.L., et al., *Evaluating the cultural validity of the stressful life events screening questionnaire.* Violence Against Women, 2006. **12**(12): p. 1191-1213.

39.	Kopp, M.S., et al., *Measures of stress in epidemiological research.* Journal of psychosomatic research, 2010. **69**(2): p. 211-225.

40.	Elemo, A.S., S.A. Satici, and M.D. Griffiths, *The Fear of COVID-19 Scale: Psychometric Properties of the Ethiopian Amharic Version.* International Journal of Mental Health and Addiction, 2020: p. 1-12.

41.	Asante, K.O., et al., *The prevalence and correlates of suicidal behaviours (ideation, plan and attempt) among adolescents in senior high schools in Ghana.* SSM-population health, 2017. **3**: p. 427-434.

42.	Seidu, A.-A., et al., *Suicidal behaviours among in-school adolescents in Mozambique: Cross-sectional evidence of the prevalence and predictors using the Global School-Based Health Survey data.* PloS one, 2020. **15**(7): p. e0236448.

43.	Darré, T., et al., *Suicidal ideation and suicide attempts in subjects aged 15–19 in Lomé (Togo).* BMC research notes, 2019. **12**(1): p. 1-4.

44.	Ismail, I.S., K. Abdulsatar, and H.A. Baey, *Prevalence and associated factors of suicidal behavior among secondary school students in Kut City.* Medical Journal of Babylon, 2011. **8**(4): p. 561-568.

45.	Guedria-Tekari, A., et al., *Suicidal ideation and suicide attempts among Tunisian adolescents: prevalence and associated factors.* The Pan African Medical Journal, 2019. **34**.

46.	Quarshie, E.N.-B., H.K. Onyeaka, and K.O. Asante, *Suicidal behaviours among adolescents in Liberia.* BMC psychiatry, 2020. **20**(1): p. 1-12.

47.	Giru, B.W., *Prevalence and associated factors of suicidal ideation and attempt among high school adolescent students in Fitche Town, north Shoa, Oromia region, Ethiopia, 2012: Institutional based cross sectional study.* Journal of Health, Medicine and Nursing, 2016. **23**.

48.	Nagamitsu, S., et al., *Prevalence and Associated Factors of Suicidality in Japanese Adolescents: Results from a Population-based Questionnaire Survey.* 2020.

49.	Steele, M.M. and T. Doey, *Suicidal behaviour in children and adolescents part 1: etiology and risk factors.* Canadian journal of psychiatry, 2007. **52**(6): p. 21S.

50.	Adewuya, A.O. and E.O. Oladipo, *Prevalence and associated factors for suicidal behaviours (ideation, planning, and attempt) among high school adolescents in Lagos, Nigeria.* Eur Child Adolesc Psychiatry, 2020. **29**(11): p. 1503-1512.

51.	Buitron, V., et al., *Interpersonal stress and suicidal ideation in adolescence: An indirect association through perceived burdensomeness toward others.* Journal of Affective Disorders, 2016. **190**: p. 143-149.
