# Supplementary material for: Prevalence and associated factors of suicidal behavior among adolescents in Bahir Dar City, Northwest Ethiopia
Source: PLoS One. 2025 May 9;20(5):e0323387. doi: 10.1371/journal.pone.0323387 (PMC12064027; doi:10.1371/journal.pone.0323387)
Supplement: Attachment — Submitted filename: Response_to_Reviewers_auresp_2.docx [file pone.0323387.s004.docx]

**To: Sharada P Wasti, Ph.D**

**Academic Editor**

**PLOS ONE**

Thank you for considering our manuscript and for arranging for it to be reviewed by two reviewers. We have tried to address your comments and the comments / suggestions from the two reviewers. In the Rebuttal letter, we copy each of the comments in bold and provide the RESPONSE underneath. We provide a clean manuscript file version of the revised manuscript with line numbering and this is uploaded as a separate file labeled 'Clean manuscript final version'. We also provide an edited manuscript with track changes and this is uploaded as a separate file labeled as ‘Edited manuscript with Track changes’. We hope we have satisfactorily addressed all the comments and hope that our paper may now be suitable for publication in your journal.

Best wishes

Abdulbasit Sherfa

Corresponding author

**Journal Requirements:**

Please review your reference list to ensure that it is complete and correct. If you have cited papers that have been retracted, please include the rationale for doing so in the manuscript text, or remove these references and replace them with relevant current references. Any changes to the reference list should be mentioned in the rebuttal letter that accompanies your revised manuscript. If you need to cite a retracted article, indicate the article’s retracted status in the References list and also include a citation and full reference for the retraction notice.

**Response**: Thank you for your recommendations; we evaluated the reference list and attempted to complete and correct some references, such as reference numbers 5–8, 11, 24, 25, and 27 in the revised article. In addition, four references (Refs. 31, 49, 52, 53, and 54) have been added to the amended manuscript’s methods and discussion section.

**Additional Editor Comments:**

**I greatly appreciate all authors for your thorough review and making revisions to the manuscript, which has vastly improved its clarity. However, there are still some remaining concerns and areas for improvement in this manuscript. I kindly request that you carefully review and address each of the concerns indicated below before reaching a final decision**

**Abstract section:**

**Background—remove “therefore” from your last sentence.**

**Response: Thank you for your comments and we removed it in the revised manuscript as follow:**

Suicide is a significant public health issue worldwide and the third leading cause of death among 15–19-year-olds. In Ethiopia, while suicidal behaviors among adolescents have a considerable impact, there is limited information on these behaviors, especially in community settings. The study aimed to assess the prevalence and associated factors of suicidal behavior among adolescents in Bahir Dar city.

**Methods: remove the date of “May 1-May 30” and keep only “May 2021”.
• Talk first about the study design, followed by the study sites, respondents, sample size and sampling technique, data collection, and analysis, which should be coherent rather than random. Your second sentence, “Mini International Neuropsychiatric Interview (MINI) was used to assess suicidal behaviors,” is not clear about what you intend to state in the current form.**

**Response: Thank you for your suggestion and comments and we tried to correct in the revised manuscript as follow:**

**Methods:** A community-based cross-sectional study was conducted in May 2021 in Bahir Dar City, Northwest Ethiopia, with 692 adolescents aged 11 to 19. Participants were chosen using a multistage sampling procedure. Face-to-face interviews were used to collect data, along with semi-structured and standardized questionnaires. Participants were considered suicidal if they replied "yes" to at least one of the six Mini International Neuropsychiatric Interview questions. The data were processed using Epi-Data version 3.1 and analyzed with the Statistical Package for Social Science version 24. Logistic regression analysis with adjusted odds ratio was used to determine the relationship between outcome and predictor variables. A p-value of <0.05 was considered statistically significant.

**• Results: Correct your way of presenting of inside small bracket (AOR XX, 95%CI xx to xx) which should be like (AOR 2.17, 95%CI 1.07 to 4.40) and make it consistent in the entire report.**

**Response: Thank you for your suggestion. We have made the necessary corrections in the revised manuscript as per your request, using small brackets. However, upon reviewing several papers published in PLOS ONE, we noticed that the range of confidence intervals is often separated by a hyphen or comma rather than the word "to." To ensure consistency throughout the revised manuscript, we have opted to use commas and we corrected it as follow:**

**Results:** The prevalence of suicidal behavior was found to be 19.8% with 95% CI (16.6, 22.8). Regarding predictive variables: low social support (AOR = 12.42, CI = 5.95, 25.95), expressed more anger (AOR = 7.30, CI = 2.51, 21.22), negative childhood experiences (AOR = 7.11, CI = 3.56, 14.20), family history of suicidal attempts (AOR = 5.66, CI = 2.44, 13.10), have no a good relationship with parents (AOR = 5.59, CI = 2.59, 12.08), depression (AOR = 5.32, CI = 2.64, 10.70), stressful life events (AOR = 2.91, CI = 1.45, 4.91), and interpersonal stress (AOR = 2.17, 95% CI 1.07, 4.40) were significantly increased odds of suicidal behavior among respondents.

**• Remove “recommendation” from your conclusion and recommendation; keep only the conclusion in the section heading and follow at the end of your conclusion section as well.**

**• Recommendation sentences should be pragmatic to combat the burden**

**Response: Thank you for your suggestion and we tried to correct it in the revised manuscript and also we tried to make our recommendation pragmatic to combat the burden of suicidal behavior among adolescents as follows:**

**Conclusion:** The prevalence of suicidal behavior was higher in this study area. Depression, stressful life events, poor parental relationships, negative childhood experiences, high anger expression, low social support, and a family history of suicidal attempts were the significant contributors. As a result, mental health interventions should focus on providing psychosocial support to adolescents, improving the relationship between adolescents and their parents by raising parents' awareness of adolescent mental health, and incorporating youth-friendly health services that promote mental well-being. Policymakers can also consider making mental health education mandatory in schools.

**Introduction section:**

**• Define first-time use abbreviation, i.e. WHO**

**Response: We have corrected it in the revised manuscript and defined it as 'World Health Organization.'"**

**Methods and material section:**

**• The study design section should be clearly articulated using credible sources to back up your claims.**

**Response: Thank you for your feedback; we attempted to look into the study design section and modified it in the revised version, as follows:**

**In May 2021, a community-based cross-sectional survey was carried out in Bahir Dar City, Northwest Ethiopia. The method was used to assess the prevalence of suicide behaviors and associated factors in the population at a specific point in time. Cross-sectional studies are frequently employed in public health research to assess the prevalence of health problems and their factors (1).**

**• Under the study setting, explain the distinction between “comprehensive specialized and specialized hospitals” in Ethiopia.**

**Response: We have explained the distinction between “comprehensive specialized and specialized hospitals” in Ethiopia in the revised manuscript on the study setting part as follows:**

There are three public hospitals in the city: one primary, one specialized, and one comprehensive. While specialized hospitals concentrate on a single area of healthcare and treat cases within their field, comprehensive specialized hospitals in Ethiopia offer a wider variety of advanced services across many specializations and handle more difficult and diversified cases. In addition, the city has four private hospitals. Inpatient psychiatric cares are only offered by comprehensive specialized and specialized public institutions

**• Sample size determination and sampling methods section: delete the word “determination” and make the section title "Sample size and sampling methods.". If you keep the sample size calculation formula, which you did in your previous version, that would be perfect to explain how 692 was calculated.**

**Response: Thank you for your suggestion. We removed the word “determination” and we made the section title "Sample size and sampling methods” in the revised manuscript as follows:**

**Sample size and sampling methods**

The sample size was determined with a single population proportion formula, based on a previous reported suicide behavior prevalence of 22.5% from a study done in Ethiopia (2). A 95% confidence interval, a 4% margin of error, and a design effect of 1.5 were employed.

Sample size was calculated as: N=Z_α/2_ × P (1-P)/W^2^_._

Where Z_α/2_ = confidence level (1.96) at CI of 95%

N= Sample size

P=22.5% (proportion of adolescents who have suicidal ideation)

W= margin of sampling error.

1- P= sample error proportion of adolescents who have no suicidal ideation

N = (1.96)² (0.225) (1-0.225) / (0.04)^2^ = 419, then multiply by 1.5 for design effect, which gives 629. By assuming a 10% non-response rate, the final sample size was 692.

The multistage sampling method was employed to choose three sub-cities from a total of six and to choose six corresponding administrative kebeles (the smallest administrative entity) from a total of seventeen. The households in the administrative kebeles were selected by systematic random sampling technique after identifying an initial starting household by use of a random number. Eligible adolescents in the selected household were further selected and interviewed. Only one adolescent member of the household was selected by the lottery method for the interview on suicide behavior.

**• Add methods and tools under the “data collection” section, and the section title should be "Data collection methods and tools."**

**Response: We have made necessary corrections to the title and also included the data quality control section in data collection methods and tools in the revised manuscript as follows:**

**Data collection methods and tools**

The questionnaire included items to assess socio-demographic information, suicidal behavior, self-esteem, social support, adverse childhood experience, anger expression, stressful life events, clinical factors, behavioral factors, and interpersonal stress.

To ensure the data quality, the questionnaire was first developed in English, translated into Amharic language, and translated back into English by different experts to check its consistency. The data was collected using an interviewer-administered questionnaire from the selected household by three health extension workers, one BSc psychiatry nurse supervisor, and the principal investigator engaging in the supervision.

The data collectors and the supervisor were given one-day training by the principal investigator. A pre-test was carried out on 5% of the respondents (35 adolescents) in Woreta town, and according to the pre-test, there was no need for a modification for the questionnaire. The supervisor and principal investigator were closely following the day-to-day data collection process for completeness, clarity, and consistency on a daily basis.

**• Remove “measurement” and add tools, and your section title looks like “Data collection tools.”**

**Response: We replaced “measurement” by “Data collection tools”**

**• Remove the separate sub-section of “data quality control” and use key quality control measures information in the above Data Collection Methods and Tools section.**

**Response: we removed the separate sub-section of “data quality control” and put under data collection method section in the revised manuscript as follow:**

The questionnaire included items to assess socio-demographic information, suicidal behavior, self-esteem, social support, adverse childhood experience, anger expression, stressful life events, clinical factors, behavioral factors, and interpersonal stress.

To ensure the data quality, the questionnaire was first developed in English, translated into Amharic language, and translated back into English by different experts to check its consistency. The data was collected using an interviewer-administered questionnaire from the selected household by three health extension workers, one BSc psychiatry nurse supervisor, and the principal investigator engaging in the supervision.

The data collectors and the supervisor were given one-day training by the principal investigator. A pre-test was carried out on 5% of the respondents (35 adolescents) in Woreta town, and according to the pre-test, there was no need for a modification for the questionnaire. The supervisor and principal investigator were closely following the day-to-day data collection process for completeness, clarity, and consistency on a daily basis.

**• Remove “processing” and keep only Data analysis.**

**Response: we removed “processing” and made it as per your request “Data analysis”**

**In the revised manuscript as follow:**

**Data analysis**

The data was entered into Epi data version 3.1 and exported to SPSS version 24 for analysis. Necessary data processing like recoding, categorizing, merging, computing, and counting was done before the actual data analysis. After data processing, descriptive statistics like measures of central tendency (mean, median, and mode) and measures of dispersion were used for continuous variables and frequency count and proportion were used to summarize categorical variables. All variables with a p-value of less than 0.2 in the bivariate logistic regression analysis were entered into the multivariable logistic regression model to identify factors associated with suicidal behavior. The adjusted odds ratios (AORs) with 95% confidence intervals were used to assess the strength of associations between the outcome and predictor variables. The p-value of <0.05 was considered significant.

**• What was the reason for including a bivariate analysis of a p-value of less than 0.2 in multivariable logistic regression analysis instead of a p-value of <0.05? Explain with credible sources to back up your argument.**

**Response:** We valued this inquiry and endeavored to elucidate the rationale for incorporating a bivariate analysis with a p-value of less than 0.2 in multivariable logistic regression analysis, rather than a p-value of less than 0.05, as follows:

Employing a p-value threshold of <0.2 in bivariate analysis, as opposed to a p-value of <0.05 in multivariable logistic regression, is grounded on statistical and practical considerations that facilitate the formation of a robust explanatory model.

Initially, employing a strict threshold of p-value < 0.05 in the bivariate analysis may lead to the exclusion of variables that are not statistically significant independently but could attain significance or relevance when adjusted for other variables in the multivariable model; thus, variables with p-values ranging from 0.05 to 0.2 may still be pertinent factors. Secondly, a variable might act as a confounder, so if we use a p-value < 0.05, it might result in the exclusion of such confounding variables, which in turn leads to biased estimates in the multivariable model. Thirdly, in many studies, especially exploratory or observational research, the goal is to identify all possible predictors of the outcome, even if their individual significance is marginal (3, 4).

Additionally, employing a p-value of <0.2 instead of the stringent threshold is significant for considerations of statistical power. This means in smaller datasets like ours, the ability to detect a true effect (power) is limited, so using a stricter threshold (p < 0.05) might fail to detect variables that are truly associated with the outcome but have smaller effect sizes. While all these mentioned reasons would balance errors, that means reducing the risk of Type II errors (missing important variables) at the cost of a slightly higher risk of Type I errors (including irrelevant variables, which is often acceptable in the early stages of model building) (4, 5).

**Results
• Proofread and state “Results”**

**Response: We tried to correct it in the revised manuscript.**

**• Make sure the small and capital letters of section sub-headings are constant across the report, such as “Socio-demographic Characteristics of the Respondents,” which are inconsistent across the section.**

**Response: We tried to correct it in the revised manuscript.**

**• In a quantitative paper, the best way is to use respondents instead of participants and maintain consistency.**

**Response: Thank you for your suggestion. To be consistent, we used respondents instead of participants in the revised manuscript.**

**• Check the entire writing and thoroughly proofread, i.e., after the full stop in Table 1, see Tables 2 and 3, which are inconsistent and require careful proofreading.**

**Response: We have made corrections in the revised manuscript to ensure that the writing of our results is consistent throughout.**

**• Refer to Figures 1 and 2 individually rather than in the same location. So, describe the first results and signpost that figure, then put the second figure's content and figure number on the signpost.**

**Response:** We accepted this comment and tried to correct it in the revised manuscript.

**• Correct the Y-axis of both of your figures, i.e., is this a percentage or the number of responders, which should be a percentage based on your data? I would recommend keeping only Figure 2 in the body of content and removing Figure 1.**

**Response: thank you for your suggestion. I have removed Figure One and made corrections to the y-axis of Figure 2 and made it Figure 1 in the revised manuscript. While revising the the manuscript, the figure files was uploaded to the Preflight Analysis and Conversion Engine (PACE) digital diagnostic tool and ensured that figures meet PLOS requirements.**

The study revealed a prevalence of suicidal behavior at 19.8% (125 individuals), with a 95% confidence interval ranging from 16.6 to 22.8. Almost half (48%) of the respondents were late adolescents (ages 18–19), and a significant majority (71.2%) were female. Among female respondents, 25.6% reported experiencing suicidal behavior, while 74.4% indicated they had not. Furthermore, 34% reported a family history of suicide attempts, 85% had experienced adverse childhood events, and 22.4% had a history of alcohol use. Almost half (48%) indicated poor relationships with their parents, while 76% reported experiencing stressful life events. Regarding anger expression, 53.6% of participants exhibited moderate levels, while 33.6% displayed high levels. Among those with low anger expression, 89.3% indicated they had not engaged in suicidal behavior, whereas 10.7% did report such behavior. In comparison, only 51.7% of individuals with high anger expression reported no suicidal behavior, with 48.3% admitting to it (refer to Figure 1).

**• Keep only “Factors Associated with Suicidal Behaviour among Adolescents” and remove “participant” from the section sub-section heading. Remove the first paragraph's information, which should be clearly articulated in your data analysis section, not under the findings section.**

**• The overall findings section should be succinct and clearly describe the findings without providing any methodological details.**

**Response: Thank you for your comments we tried to correct them in the revised manuscript. We removed the first paragraph information and also removed “participant” and kept only “Factors associated with suicidal behavior among adolescents”.**

**Factors associated with suicidal behavior among adolescents**

In the multivariate analyses, family history of suicidal attempts, facing stressful life experiences, having adverse childhood experiences, having poor social support, not having a good relationship with their parents, depression, interpersonal stress, and having high anger expression behaviors were found to be significantly associated with suicidal behavior at p < 0.05.

Adolescents with poor social support were approximately 12.42 times more likely (AOR = 12.42, CI = 5.95, 25.95) to report suicidal behavior compared to their peers who had strong social support. Additionally, those who exhibited high levels of anger expression had 7.3 times the odds of reporting suicidal behavior (AOR = 7.30, CI = 2.51, 21.22) compared to individuals with lower anger expression. Furthermore, adolescents who experienced adverse childhood events, such as abuse, neglect, and household dysfunction, faced a sevenfold increase in the risk of suicidal behavior (AOR = 7.11, CI = 3.56, 14.20) compared to their peers.

Moreover, adolescents with a family history of suicide attempts had a 5.66 times higher risk (AOR = 5.66, CI = 2.44, 13.10) of exhibiting suicidal behavior than those without such a background. Participants who reported poor relationships with their parents also showed increased odds (AOR = 5.59, CI = 2.59, 12.08) of developing suicidal behavior compared to those with positive parental relationships. Those suffering from depression were about five times more likely (AOR = 5.32, CI = 2.64, 10.70) to engage in suicidal behavior. Similarly, adolescents facing stressful life experiences had higher odds (AOR = 2.91, CI = 1.45, 4.91) of suicidal behavior compared to their peers. Finally, participants experiencing high levels of interpersonal stress were approximately twice as likely (AOR = 2.17, 95% CI = 1.07, 4.40) to report suicidal behavior compared to those with lower levels of interpersonal stress. (Table 3)

**Discussion section:**

**• Outline the first paragraph of what you are going to discuss in this section, which I gave you last suggestions but did not address yet. Your discussion should outline two sections, i.e., the prevalence of suicidal behaviour and factors associated with suicidal behaviour, and critically articulate as per the last provided suggestions.**

**Response: we have made necessary correction based on your suggestion in the revised manuscript as follow:**

# Discussion

The main objective of this study was to assess the prevalence and factors influencing suicidal behavior among adolescents living in Bahir Dar city. The results revealed that the prevalence of suicidal behavior was 19.8% (95% CI: 16.6, 22.8). Key factors associated with suicidal behavior included a family history of suicide, exposure to stressful life events, adverse childhood experiences, poor social support, troubled parent-child relationships, depression, and high levels of anger expression. This study emphasizes that suicidal behavior is a significant public health issue for adolescents living in Bahir Dar city.

**Prevalence of suicidal behaviors**

This finding was consistent with a study conducted in Ethiopia among high school students, which reported that 16.2% had suicidal attempts and 22.5% had suicidal ideation (2). Additionally, the result matched those studies done in Ghana among high school students, which found that 18.2% had suicidal thoughts, 22.5% had suicidal plans, and 22.2% had attempted suicide (5); in Mozambique, where 17.7% had suicidal ideation and 18.5% had attempted suicide (6); and in Togo (16.5% had suicidal thoughts) (7).

However, the finding in this study was higher than the school-based study done in Kut City, which reported suicidal behavior to be 8.3% (8) and Tunisia (9.6% suicidal ideation and 7.3% suicidal attempt) (9). On the contrary, the finding was lower than studies done in Liberia, where the prevalence of suicidal attempts and suicidal ideation were 33.7% and 26.8% among adolescent students, respectively (10), Lebanese suicidal ideation was 28.9% (11), and Benin (23.2% suicidal thought and 28.3% suicidal attempt) (12). The discrepancy might result from variations in the socio-demographic, economic, and cultural attributes of respondents, sample size, and study context, as well as differences in the measurement instruments employed; this study utilized the Mini International Neuropsychiatric Interview (MINI) Suicidal Scale (13).

**Factors associated with suicidal behavior**

In our study, suicidal behavior was significantly associated with a family history of suicide, stressful life experiences, adverse childhood experiences, poor social support, poor parent-child interactions, depression, and high anger-out expressive behavior. Adolescents with low social support had a 12.42 times higher risk of suicidal behavior than those with good social support (AOR=12.42, CI=5.95, 25.95). Similarly, previous research in Ethiopia demonstrated a relationship between low social support and suicidal behavior (2, 14, 15). The correlation may arise from the fact that being neglected by family and friends and not getting instrumental, informational, and emotional support from important others greatly increases feelings of worthlessness and hopelessness, which in turn contribute to suicidal behavior (16).

Adolescents who did not have a good relationship with their parents were 5.59 times more highly at risk for suicidal behavior than those who had a good relationship with their parent (AOR=5.59, 95% CI= 2.59, 12.08). These findings were supported by prior research conducted in China and Japan (17, 18). Adolescents who did not have a good relationship with their parents were 5.59 times more highly at risk for suicidal behavior than those who had a good relationship with their parent (AOR=5.59, 95% CI= 2.59, 12.08). These findings were supported by prior research conducted in China and Japan

The current study discovered that adolescents with higher anger expression behavior had approximately seven times (AOR=7.30, CI= 2.51, 21.22) the odds of reporting suicidal conduct as those with lower anger expression behavior. The possible reason may be individuals with anger-related disorders exhibit a range of cognitive and interpersonal deficiencies that have a negative impact on well-being and contribute to a higher risk for suicide behaviors, such as poor social problem-solving abilities and poor social reasoning. The findings of this study lend support to the concept that high levels of external rage emotions are significantly related to depressive symptoms and suicidal conduct.

Adolescent having adverse child-hood experience (abuse, neglect and household dysfunction) had seven fold (AOR=7.11, CI= 3.56-14.20) higher risk of having suicidal behavior compared to their counterparts Previous studies also found that adolescents who experience abuse, physical hurt, and violence are positively associated with suicidal behavior (19). Adolescents who had to experience stressful life events had 2.91 times the odds of suicidal behavior than their counterparts [(AOR= 2.91, 95% CI=1.43, 5.90)]. This finding was supported by previous studies in which suicidal behavior was significantly favored by stressful life events (9, 20). Adverse childhood experiences and stressful life events may result in enduring psychological effects, heightening susceptibility to mental health disorders such as depression and anxiety, or exacerbating pre-existing conditions, thereby increasing suicidal ideation and behaviors (21-23).

Respondents who had to experience stressful life events had 2.91 times the odds of suicidal behavior than their counterparts (AOR= 2.91, 95% CI=1.43, 5.90). This finding was supported by previous studies in which suicidal behavior was significantly favored by stressful life events (9, 20). Adverse childhood experiences and stressful life events can lead to lasting psychological effects, including increased vulnerability to mental health issues like depression and anxiety, or can trigger or worsen existing mental health issues, leading to increased suicidal thoughts and behaviors.

Adolescents with a family history of suicidal conduct were 5.66 times more likely to attempt suicide than adolescents without such a history (AOR = 5.66, 95% CI 2.44, 13.10). This finding was supported by a study done in Fitche Town, North Shoa, Oromia region (14), but no significant associations were observed in the study conducted in Dangila town (2). The discrepancy could be attributed to the study subjects' coping mechanisms, as well as the sample size and study context.

Respondents with depression showed a fivefold higher risk of suicide conduct (AOR = 5.32, 95% CI = 2.64, 10.70) than those without depression. Previous studies (8, 24, 25) have also found that adolescents who have depression are positively associated with suicidal behaviors. Participants who had high interpersonal stress had approximately 2 times the odds (AOR=2.17, 95% CI= 1.07, 4.40) of suicidal behavior compared to those who had low interpersonal stress. A previous study also supports that high interpersonal stress was significantly associated with suicidal behavior (26). The possible reason for this might be Stress in interpersonal domains, meaning stress that occurs in the context of relationships with others, has consistently been identified as a significant correlate or predictor of suicidal behavior in adolescence.

**• Carefully review your strengths and limitations section and make it very clear and correct statements instead of very generic statements.**

**Response: Thank you for your suggestion; we revised it as follows:**

**Strength and limitation of the study**

# This study was not without limitations. First and foremost, because the study is cross-sectional, causal relationships between independent factors and suicide behaviors cannot be explained. Second, due to the sensitive nature of the topic, there may have been reporting bias during data collection. Third, the study failed to account for the prevalence of other mental or psychological problems besides depression, which could have been linked to suicidal behavior. Furthermore, this study was unable to evaluate the techniques of suicide attempts. The study's strengths included the use of standard and validated tools to look at dependent and independent variables, evaluation of characteristics concerning the COVID-19 epidemic, and being a community-based study.

**Conclusion section:**

**• Remove “and recommendation” and keep only the conclusion of the section title.
• Provide pragmatic recommendations to address the burden instead of generic and vague statements.**

**Response: we removed recommendation” and made it the title only conclusion in the revised manuscript and tried to make recommendation pragmatic to address the burden as follows:**

**Conclusion**

The prevalence of suicide behavior was rather high in this study, indicating a substantial public health risk among teenagers in Bahir Dar that demands special attention. Suicidal behavior was significantly associated with a lack of social support, depression, a family history of suicide, not having a good relationship with their parent, adverse childhood experiences, and experiencing stressful live events**.** The findings suggest that mental health interventions need to be implemented within communities to enhance the relationship between parents and adolescents through raising parental awareness of adolescent mental health and integrating youth-friendly health services that promote adolescent mental well-being and offer psychosocial support. Policymakers may also consider instituting a required mental health program in schools.

**Proofreading is required to enhance writing patterns and ensure consistency, and this should be reflected in your track change version. Several sections i.e. inconsistent small and capital letters, table/figure notations and using notations after a full stop**

**Response: we tried to check the entire manuscript and made it consistent.**

1.	Gordis L. Epidemiology. 5th ed: Elsevier Saunders; 2014.

2.	Amare T, Meseret Woldeyhannes S, Haile K, Yeneabat T. Prevalence and associated factors of suicide ideation and attempt among adolescent high school students in Dangila town, Northwest Ethiopia. Psychiatry journal. 2018;2018.

3.	Hosmer Jr DW, Lemeshow S, Sturdivant RX. Applied logistic regression: John Wiley & Sons; 2013.

4.	Mickey RM, Greenland S. The impact of confounder selection criteria on effect estimation. American journal of epidemiology. 1989;129(1):125-37.

5.	Asante KO, Kugbey N, Osafo J, Quarshie EN-B, Sarfo JO. The prevalence and correlates of suicidal behaviours (ideation, plan and attempt) among adolescents in senior high schools in Ghana. SSM-population health. 2017;3:427-34.

6.	Seidu A-A, Amu H, Dadzie LK, Amoah A, Ahinkorah BO, Ameyaw EK, et al. Suicidal behaviours among in-school adolescents in Mozambique: Cross-sectional evidence of the prevalence and predictors using the Global School-Based Health Survey data. PloS one. 2020;15(7):e0236448.

7.	Darré T, Consuela KAC, Saka B, Djiwa T, Ekouévi KD, Napo-Koura G. Suicidal ideation and suicide attempts in subjects aged 15–19 in Lomé (Togo). BMC research notes. 2019;12(1):1-4.

8.	Ismail IS, Abdulsatar K, Baey HA. Prevalence and associated factors of suicidal behavior among secondary school students in Kut City. Medical Journal of Babylon. 2011;8(4):561-8.

9.	Guedria-Tekari A, Missaoui S, Kalai W, Gaddour N, Gaha L. Suicidal ideation and suicide attempts among Tunisian adolescents: prevalence and associated factors. The Pan African Medical Journal. 2019;34.

10.	Quarshie EN-B, Onyeaka HK, Asante KO. Suicidal behaviours among adolescents in Liberia. BMC psychiatry. 2020;20(1):1-12.

11.	Pengpid S, Peltzer K. Suicide attempt and associated factors among adolescents in five Southeast Asian countries in 2015. Crisis: The Journal of Crisis Intervention and Suicide Prevention. 2020.

12.	Randall JR, Doku D, Wilson ML, Peltzer K. Suicidal behaviour and related risk factors among school-aged youth in the Republic of Benin. PLoS one. 2014;9(2):e88233.

13.	Roaldset JO, Linaker OM, Bjørkly S. Predictive validity of the MINI suicidal scale for self-harm in acute psychiatry: a prospective study of the first year after discharge. Archives of suicide research. 2012;16(4):287-302.

14.	Giru BW. Prevalence and associated factors of suicidal ideation and attempt among high school adolescent students in Fitche Town, north Shoa, Oromia region, Ethiopia, 2012: Institutional based cross sectional study. Journal of Health, Medicine and Nursing. 2016;23.

15.	Abdu Z, Hajure M, Desalegn D. Suicidal behavior and associated factors among students in Mettu University, South West Ethiopia, 2019: an institutional based cross-sectional study. Psychology research and behavior management. 2020;13:233.

16.	Park S-M, Cho S-i, Moon S-SJJopr. Factors associated with suicidal ideation: role of emotional and instrumental support. 2010;69(4):389-97.

17.	Liu X, Chen H, Liu Z, Wang J, Jia C. Prevalence of suicidal behaviour and associated factors in a large sample of Chinese adolescents. Epidemiology and psychiatric sciences. 2019;28(3):280-9.

18.	Nagamitsu S, Mimaki M, Koyanagi K, Tokita N, Kobayashi Y, Hattori R, et al. Prevalence and Associated Factors of Suicidality in Japanese Adolescents: Results from a Population-based Questionnaire Survey. 2020.

19.	Murphy A, Steele M, Dube SR, Bate J, Bonuck K, Meissner P, et al. Adverse childhood experiences (ACEs) questionnaire and adult attachment interview (AAI): Implications for parent child relationships. Child abuse & neglect. 2014;38(2):224-33.

20.	Steele MM, Doey T. Suicidal behaviour in children and adolescents part 1: etiology and risk factors. Canadian journal of psychiatry. 2007;52(6):21S.

21.	Cluver L, Orkin M, Boyes ME, Sherr L. Child and adolescent suicide attempts, suicidal behavior, and adverse childhood experiences in South Africa: A prospective study. Journal of Adolescent Health. 2015;57(1):52-9.

22.	Miller AB, Eisenlohr-Moul T, Giletta M, Hastings PD, Rudolph KD, Nock MK, et al. A within-person approach to risk for suicidal ideation and suicidal behavior: Examining the roles of depression, stress, and abuse exposure. Journal of consulting and clinical psychology. 2017;85(7):712.

23.	Turecki G, Brent DA. Suicide and suicidal behaviour. The Lancet. 2016;387(10024):1227-39.

24.	Adewuya AO, Oladipo EO. Prevalence and associated factors for suicidal behaviours (ideation, planning, and attempt) among high school adolescents in Lagos, Nigeria. European child & adolescent psychiatry. 2020;29(11):1503-12.

25.	Pandey AR, Bista B, Dhungana RR, Aryal KK, Chalise B, Dhimal M. Factors associated with suicidal ideation and suicidal attempts among adolescent students in Nepal: Findings from Global School-based Students Health Survey. PloS one. 2019;14(4):e0210383.

26.	Buitron V, Hill RM, Pettit JW, Green KL, Hatkevich C, Sharp C. Interpersonal stress and suicidal ideation in adolescence: An indirect association through perceived burdensomeness toward others. Journal of Affective Disorders. 2016;190:143-9.
