# Supplementary material for: Prevalence and associated factors of suicidal behavior among adolescents in Bahir Dar City, Northwest Ethiopia
Source: PLoS One. 2025 May 9;20(5):e0323387. doi: 10.1371/journal.pone.0323387 (PMC12064027; doi:10.1371/journal.pone.0323387)
Supplement: Attachment — Submitted filename: Responses to Reviewers.docx [file pone.0323387.s005.docx]

**To: Sharada P Wasti, Ph.D**

**Academic Editor**

**PLOS ONE**

Thank you for considering our manuscript. We have tried to respond to your comments and recommendations. In the response letter, we bold each of the comments and include the RESPONSE beneath. We give a clean manuscript file version of the amended manuscript with line numbering, which is submitted as a separate file named 'Manuscript.' We also include an amended manuscript with track changes, which is uploaded as a separate file titled 'Revised manuscript with track changes.' We belief we sufficiently addressed all comments and are pleased that our manuscript is now suitable for publication in your journal.

Best wishes

Abdulbasit Sherfa

Corresponding author

**Journal Requirements:**

**Please review your reference list to ensure that it is complete and correct. If you have cited papers that have been retracted, please include the rationale for doing so in the manuscript text, or remove these references and replace them with relevant current references. Any changes to the reference list should be mentioned in the rebuttal letter that accompanies your revised manuscript. If you need to cite a retracted article, indicate the article’s retracted status in the References list and also include a citation and full reference for the retraction notice.**

**Response**: Thank you for your recommendations; we evaluated the reference list and attempted to complete and correct some references.

**Additional Editor Comments:**

I greatly appreciate all authors for your thorough review and making revisions to the manuscript, which has vastly improved its clarity. However, there are still some remaining concerns and areas for improvement in this manuscript. I kindly request that you carefully review and address each of the concerns indicated below before reaching a final decision:

**Abstract section:**

**• Methods section: Present clearly and concisely and also add the suicidal measurement tool instead of grading criteria. Clear and concise, this sentence: The data were processed using Epi-Data version 3.1 and analysed with the Statistical Package for Social Science version 24.**

**Response: Thank you for your suggestion and we tried to make it more concise and clear on the revised manuscript as follows:**

**Methods**: A community-based cross-sectional study involving 692 adolescents was conducted in Bahir Dar City, Northwest Ethiopia, in May 2021. Participants were selected through a multistage sampling technique. Semi-structured and standardized questionnaires were employed to gather data via face-to-face interviews. Suicidal behavior was evaluated using the six questions of the Mini International Neuropsychiatric Interview. Epi-Data version 3.1 was utilized for data entry, whereas Statistical Package for Social Science version 24 was employed for analysis. Logistic regression with an adjusted odds ratio determined the relationship between outcome and factor variables. A p-value of <0.05 was considered statistically significant.

**• Findings section: Look at the last feedback in your findings section. The presentation should be succinct with a small bracket (AOR XX, 95% CI XX to XX), which should be like (AOR 2.17, 95% CI 1.07 to 4.40), and make it consistent in the entire report. But it still has not been fixed in the abstract section. So make the consistency of your findings presentations, i.e., at (AOR 7.30, 95% CI 2.51 to 21.22), negative childhood experiences (AOR 7.11, 95% CI 3.56 to 14.20) to all abstract and FINDINGS sections for the consistency with neat and clean presentation.**

**Response**: **Thank you for your suggestion and we tried to correct it in the revised manuscript as follows:**

**Results:** The prevalence of suicidal behavior was found to be 19.8% with 95% CI (16.6, 22.8). Regarding predictive variables: low social support (AOR 12.42, 95% CI 5.95 to 25.95), expressed more anger (AOR 7.30, 95% CI 2.51 to 21.22), negative childhood experiences (AOR 7.11, 95% CI 3.56 to 14.20), family history of suicidal attempts (AOR 5.66, 95% CI 2.44 to 13.10), have no a good relationship with parents (AOR 5.59, 95% CI 2.59 to 12.08), depression (AOR 5.32, 95% CI 2.64 to 10.70), stressful life events (AOR 2.91, 95% CI 1.45 to 4.91), and interpersonal stress (AOR 2.17, 95% CI 1.07 to 4.40) were significantly increased odds of suicidal behavior among respondents.

**• Conclusion section: make the section very clear and concise.**

**Response: Thank you for your suggestion and we tried to make it more concise and clear on the revised manuscript as follows:**

**Conclusion:** Suicidal behavior was prevalent in the study area, and factors such as depression, stressful life events, poor parental relationships, negative childhood experiences, high anger expression, low social support, and a family history of suicidal attempts were the significant contributors. Mental health interventions have to emphasize psychosocial support for adolescents, improve the parent-adolescent relationship by increasing parental knowledge about adolescents’ mental health, and integrate youth-friendly health services that foster mental well-being. Policymakers can also propose mandating mental health guidance in schools.

**• Remove the corresponding author's phone number, i.e., Phone: +251-913-69-28-80.**

**Response**: Thank you for the comment, the phone number from corresponding is removed and corrected as follows

****Correspondent Author:*** *Abdulbasit Sherfa, E-mail: abdusherfa3@gmail.com*

Findings section:

**• All table titles should be short and clear as per the last feedback and made consistent, i.e., Table 1:, Table 2., and Table 3.**

**Response: Thank you for your suggestion and we have made the necessary correction on the revised manuscript as follows**

*Table 1: Socio-demographic characteristics of participants*

*Table 2:* *The* *frequency distribution of participants’ psychosocial and substance-related factors*

*Table 3: Bi-variable and multivariable logistic regression of suicidal behavior and associated factors among participants*

**• The title of Figure 1 should be short and clear, i.e., Figure 1: Distribution of suicidal behaviour by level of anger expression**

**Response: Thank you for your suggestion and we have made the necessary correction on the revised manuscript as follows**

*Figure 1: Percentage distribution of suicidal behavior by level of anger expression*

**• Table two's title should be shorter as per earlier feedback, and category variables need to be made consistent where category variables are inconsistent with other tables, i.e., the first letters are small letters, so make it consistent with other tables.**

**Response: Thank you for the comment; we have made the necessary correction on the revised manuscript as follows**

*Table 2:* *The* *frequency distribution of participant’s psychosocial and substance-related factors*

| **Variable** | **Category** | **Frequency** | **Percentage** |
| --- | --- | --- | --- |
| Family history of suicide attempt | Yes  No | 85  546 | 13.5  86.5 |
| Family history of suicidal committed | Yes  No | 2  629 | 0.3  99.7 |
| Grow up with your biological family | Yes  No | 503  128 | 79.7  20.3 |
| Relationship with parent | Good to very good  Not good/disturbed or very disturbed | 542  89 | 85.9  14.1 |
| Relationship with peers | Good  Poor | 629  2 | 99.7  0.3 |
| Social support | Poor  Moderate  Strong | 251  300  80 | 39.8  47.5  12.7 |
| Ever used tobacco/ cigarettes | Yes  No | 28  603 | 4.4  95.6 |
| In the past three month have you used tobacco/cigarettes | Yes  No | 23  608 | 3.6  96.4 |
| In your life, have you Ever used alcohol | Yes  No | 58  573 | 9.2  90.8 |
| in the past three month have you used alcohol | Yes  No | 50  581 | 7.9  92.1 |
| Ever use of khat | Yes  No | 32  599 | 5.1  94.9 |
| Khat use in the past three month | Yes  No | 26  605 | 4.1  95.9 |

**• Figure 1 removes the table and provides the % value at the top of the bar diagram and removes the below tables and keeps the legend.**

**Response: Thank you for the comment; we have made the necessary correction on the revised manuscript as follows**

|  | No | yes |
| --- | --- | --- |
| Low | 89.3 | 10.7 |
| Moderate | 83 | 17 |
| High | 51.7 | 48.3 |

*Figure 1: Percentage distribution of suicidal behaviour by level of anger expression*

- **The discussion section should be thoroughly reviewed and succinctly presented without repeating all findings with 95% CI values, but you can use AOR only and compare and contrast with global and national empirical evidence and provide your thoughts on society.**

**Response: we have made necessary correction based on your suggestion in the revised manuscript as follow:**

# Discussion

The primary objective of this study was to evaluate the prevalence and factors influencing suicidal behavior among adolescents residing in Bahir Dar city. The finding indicated that the prevalence of suicidal behavior was 19.8% (95% CI: 16.6, 22.8). Key factors associated with suicidal behavior included a family history of suicide, exposure to stressful life events, adverse childhood experiences, poor social support, troubled parent-child relationships, depression, and high levels of anger expression. This study underscores that suicidal behavior is a significant public health concern for adolescents in Bahir Dar city.

**Prevalence of suicidal behaviors**

The present finding was in line with a study of high school students in Ethiopia that found that 22.5% had suicidal ideation and 16.2% had attempted suicide (32). Furthermore, the results were consistent with studies conducted in Ghana among high school students, which found that 18.2% had suicidal thoughts, 22.5% had suicidal plans, and 22.2% had attempted suicide (42); in Mozambique, where 17.7% had suicidal ideation and 18.5% had attempted suicide (43); and in Togo (16.5% had suicidal thoughts) (44).

However, the finding in this study was higher than the school-based study done in Kut City, which reported suicidal behavior to be 8.3% (45), and Tunisia (9.6% suicidal ideation and 7.3% suicidal attempt) (46). On the contrary, the finding was lower than studies done in Liberia, where the prevalence of suicidal attempts and suicidal ideation were 33.7% and 26.8% among adolescent students, respectively (47), Lebanese suicidal ideation was 28.9% (12), and Benin (23.2% suicidal thought and 28.3% suicidal attempt) (21). The disparity may be explained by differences in the sample size, study setting, socio-demographic, economic, and cultural characteristics of participants, as well as by the measurement tools used; the Mini International Neuropsychiatric Interview (MINI) Suicidal Scale was used in this study (33).

**Factors associated with suicidal behavior**

Suicidal behavior was 12.42 times more likely to occur in adolescents with poor social support than in those with good social support. Likewise, other studies conducted in Ethiopia showed a relationship between suicidal behavior and a lack of social support (2, 32, 48). Likewise, adolescents who did not have a good relationship with their parents were 5.59 times more highly at risk for suicidal behavior than those who had a good relationship with their parents. These findings were supported by prior research conducted in China and Japan (16, 50). These relationships may arise from the fact that being neglected by family and friends and not getting instrumental, informational, and emotional support from important others greatly increases feelings of worthlessness and hopelessness, which in turn contribute to suicidal behavior (49).

Adolescents who experienced negative childhood experiences (abuse, neglect, and household dysfunction) were seven times more likely to exhibit suicidal behavior than their peers. Previous research has revealed that adolescents who have experienced abuse, physical harm, or violence are more likely to engage in suicidal behavior (36). Adolescents who had to go through stressful events in their lives had a 2.91 times higher risk of suicidal behavior than their counterparts. This finding was supported by previous studies in which suicidal behavior was significantly favored by stressful life events (46, 51). Adverse childhood experiences and stressful life events can have long-term psychological consequences, increasing vulnerability to mental health disorders, including depression and anxiety, or intensifying pre-existing conditions, increasing suicide ideation and behavior (52-54).

Adolescents who had to go through difficult life situations had almost a threefold higher risk of suicidal behavior than their counterparts. Previous research has found that stressful life situations are much more likely to promote suicidal conduct (46, 51). Adverse childhood experiences and stressful life events can have long-term psychological consequences, including greater vulnerability to mental health concerns such as depression and anxiety, or they can trigger or worsen existing mental health difficulties, leading to an increase in suicidal thoughts and actions.

Adolescents having a family history of suicidal behavior were 5.66 times more likely to attempt suicide than those without such a history. This finding was supported by a study conducted in Fitche Town, North Shoa, Oromia region (48), while no significant relationships were discovered in Dangila town (32). The difference could be due to the study subjects' strategies for coping, sample size, and study setting.

Participants with depression had a five times higher risk of suicidal behavior than those without depression. Previous research has indicated that depression in adolescents has a positive association with suicidal behavior (22, 45, 55). Participants with high interpersonal stress had roughly twice the risk of suicidal behavior as those with low interpersonal stress. A previous study also supports that high interpersonal stress was significantly associated with suicidal behavior (56). A potential explanation for this is that depression and interpersonal stress have often been recognized as important correlations or predictors of suicide conduct in adolescents.

Finally, the current study revealed that adolescents with higher anger expression behavior were roughly seven times more likely to report suicidal behavior than those with lower anger expression behavior. Individuals with anger-related illnesses may demonstrate a variety of cognitive and interpersonal impairments that harm their well-being and contribute to a higher risk of suicidal conduct, such as poor social problem-solving ability and reasoning. The outcomes of this study offer support to the idea that high levels of external angry emotions are significantly associated with depressed symptoms and suicidal behavior.

**• After completing all revisions, please fully proofread to make it clear and coherent.**

Response: we tried to check the entire manuscript and made it consistent.

- **While revising your submission, please upload your figure files to the Preflight Analysis and Conversion Engine (PACE) digital diagnostic tool, https://pacev2.apexcovantage.com/. PACE helps ensure that figures meet PLOS requirements. To use PACE, you must first register as a user. Registration is free. Then, login and navigate to the UPLOAD tab, where you will find detailed instructions on how to use the tool. If you encounter any issues or have any questions when using PACE, please email PLOS at figures@plos.org. Please note that Supporting Information files do not need this step.**

Response: The figure is uploaded to the Preflight Analysis and Conversion Engine (PACE) digital diagnostic tool, https://pacev2.apexcovantage.com/. PACE ensured that the figures meet PLOS requirements.
